# The Properties of Cu Ions in Zeolites CuY Studied by IR Spectroscopy

**DOI:** 10.3390/molecules26154686

**Published:** 2021-08-03

**Authors:** Jerzy Podobiński, Mariusz Gackowski, Grzegorz Mordarski, Katarzyna Samson, Michał Śliwa, Dorota Rutkowska-Zbik, Jerzy Datka

**Affiliations:** Jerzy Haber Institute of Catalysis and Surface Chemistry, Polish Academy of Sciences, Niezapominajek 8, PL-30239 Krakow, Poland; ncpodobi@cyf-kr.edu.pl (J.P.); mariusz.gackowski@ikifp.edu.pl (M.G.); grzegorz.mordarski@ikifp.edu.pl (G.M.); katarzyna.samson@ikifp.edu.pl (K.S.); michal.sliwa@ikifp.edu.pl (M.Ś.); dorota.rutkowska-zbik@ikifp.edu.pl (D.R.-Z.)

**Keywords:** zeolites CuY, probe molecules CO and NO, IR spectroscopy, speciation of Cu sites

## Abstract

The properties of both Cu^2+^ and Cu^+^ ions in zeolite CuY were followed with NO and CO as probe molecules. Cu^2+^ was found to be located in S_II_, S_II*_, and S_III_ sites, whereas Cu^+^ was found in S_II_ and S_II*_ sites. The fine analysis of the spectra of Cu^2+^-NO and Cu^+^-CO adducts suggests that both in S_II_ and in S_II*_ sites two kinds of Cu cations exist. They differ in the positive charge, which may be related to the varying numbers of AlO_4_^−^ in close proximity. The experiments of NO and CO adsorption and desorption evidenced that both Cu^2+^ and Cu^+^ sites of highest positive charge bind probe molecules most strongly but activate them to a lesser extent than the Cu sites of lowest positive charge. The experiments of reduction with hydrogen evidenced that the Cu ions of higher positive charge are first reduced by hydrogen. On the other hand, Cu sites of the lowest positive charge are first oxidized by oxygen. The experiments with CuNaY zeolites of various Cu contents suggest that the first introduced Cu (at low Cu contents) created Cu^+^, which was the most neutralized by framework oxygens. Such Cu cations are the most stabilized by framework oxygens.

## 1. Introduction

Cu-containing zeolites attracted a great deal of attention because of some exceptional properties of Cu ions (especially Cu^+^) located in the zeolite matrix. Because of the strong interaction of Cu ions with framework oxygens, the positive charge of cations is neutralized to a great extent and they are able to transmit electrons to adsorbed molecules. Therefore, Cu^+^ in zeolites may activate molecules of NO or other molecules (especially organic molecules containing multiple bonds). Quantumchemical calculations evidenced that this activation is realized by π back donation of d electrons of Cu to antibonding π* orbitals of molecules (e.g., [1,2,3,4]). This is why Cu-zeolites (especially CuZSM-5) are active in NO decomposition (e.g., [5,6,7,8,9]) and reduction with NH_3_ and hydrocarbons [10,11,12,13,14,15].

As Cu species in zeolites may change the oxidation state, the Cu^2+^/Cu^+^ pairs may participate in oxidation of various molecules. Therefore, Cu-containing zeolites are catalysts in several oxidation reactions. Many of these reactions are oxidation of alcohols: ethanol [16,17,18,19] or benzyl alcohol [20,21,22,23,24,25,26,27,28]. Zeolites of faujasite type are especially useful as catalysts in such reactions due to their very open structure and large cavities which can host even bulky molecules. The oxidation of benzyl alcohol to benzaldehyde has often been studied over CuY zeolites. Tsuruya et al. [21] proposed the kinetic equation and determined the activation energy. An interesting observation was that the addition of amines changed oxidation activity: the addition of piperidine decreased and the addition of pyridine increased the activity. It was related to the strong bonding of Cu^2+^ with the amine group. It was also found that the addition of CO and H_2_ into the reaction system decreased the conversion of benzyl alcohol and changed the selectivity to benzaldehyde, which was higher for hydrogen; on the other hand, water deactivated the catalyst.

In another work, Tsuruya et al. [22] reported the gas-phase oxidation of ethanol to acetaldehyde over CuNa-Y and evidenced that Cu^2+^ ions were the active sites responsible for oxidative dehydrogenation of ethanol. 

CuY also catalyzes some other reactions, such as the synthesis of dimethyl carbonate from methanol, CO, and O_2_. This reaction was studied by Drake et al. [29], who used zeolite CuY obtained by a solid-state ion exchange reaction using a thermal treatment of a HY/CuCl mixture in He at 923 K. The oxidation state, local coordination, and bond distances of Al and Cu were determined using Al K-edge and Cu K-edge X-ray absorption spectroscopy (XAS). Complimentary information was obtained by H_2_ temperature-programmed reduction and by in-situ infrared spectroscopy. Cu-Y was active for the oxidative carbonylation of methanol, and at low reactant contact time produced dimethyl carbonate as the primary product. With increasing reactant contact time, the formation of dimethoxy methane and methylformate became important. 

Cu-containing ultrastable zeolite Y (obtained by the treatment of ultrastable zeolite Y with CuCl) was also used as a versatile, efficient, and recyclable catalyst for various Ullmann-type coupling reactions [30]. Easy to prepare and cheap, this catalytic material enabled the arylation and heteroarylation of diverse O-, N-, S-, and C-nucleophiles under ligand-free conditions while exhibiting large functional group compatibility.

CuY zeolites may be also interesting catalysts for NOx abatement (by selective catalytic reduction—SCR), together with other Cu-zeolites such as CuZSM-5, Cu-CHA, CuBEA, and CuSSZ [31], as well as with FeZSM-5 zeolites. CuY showed high conversion in NO SCR; the onset temperature for maximal conversion was ca. 300 °C. Deka et al. [31] presented an overview of the properties of Cu ions in various zeolites including CuY. 

As Cu ions in zeolites Y are important active sites for numerous reactions, their properties have been studied with various methods. The overview of these methods was presented by Deka et al. [31]. Spectroscopic methods with probe molecules were especially efficient to study the status and properties of both Cu^2+^ and Cu^+^ in CuY. An elegant IR study of properties of Cu ions interacting with probe molecules (CO and NO) was carried out by Palomino et al. [32]. Independent of this experimental investigation, quantumchemical modelling concerning the localization and properties of Cu ions in zeolites CuY has been conducted [33,34]. 

This study is the continuation of our earlier investigation of the properties of Cu species in zeolites CuHFAU and CuNaFAU (of Si/Al = 31 obtained by dealumination zeolite Y of Si/Al = 2.5) in which Cu was introduced by impregnation of zeolites HFAU and NaFAU [35]. It was found that the nature of Cu species depended on Cu content and on the parent zeolite: HFAU or NaFAU. In CuHFAU most of the Cu was in the form of exchanged Cu^+^. They replace H^+^ and neutralize the aluminosilicate framework. In CuNaFAU most of the Cu was either as Cu^2+^ or in oxide form (CuO) and Cu^+^ in the form of oxide. These Cu forms had varying susceptibility for reduction with hydrogen. Cu^2+^ and Cu^+^ oxide were found to be the most prone to reduction [36]. The various forms of Cu also had different reactivities for ethanol oxidation in situ in an IR cell without oxygen supply [19]. Zeolite CuHFAU containing only protonic sites and also Cu^+^ in cationic exchange form catalyzed only ethanol dehydration to ethene. Ethene was bonded to Cu^+^, and C=C stretching which was IR inactive in the free molecule became IR active, due to the change of symmetry of the molecule bonded to the Cu^+^ site. The C=C stretching frequency was significantly lower than in the free molecule, because ethene acted as a π-acceptor ligand. Zeolite CuNaFAU primarily containing Cu in oxide forms caused the oxidation of ethanol to acetaldehyde and subsequently to acetic acid. Most probably both Cu^+^ and Cu^2+^ in oxide forms were oxygen donors in acetaldehyde formation, whereas only Cu^+^ oxide was an oxygen donor in acetaldehyde oxidation to acetic acid.

In this study, we investigated copper species in zeolite CuY of Si/Al = 2.5 using CO and NO adsorption experiments. 

## 2. Results

### 2.1. The Properties of Cu^2+^ in Zeolite CuY

**Figure 1:**
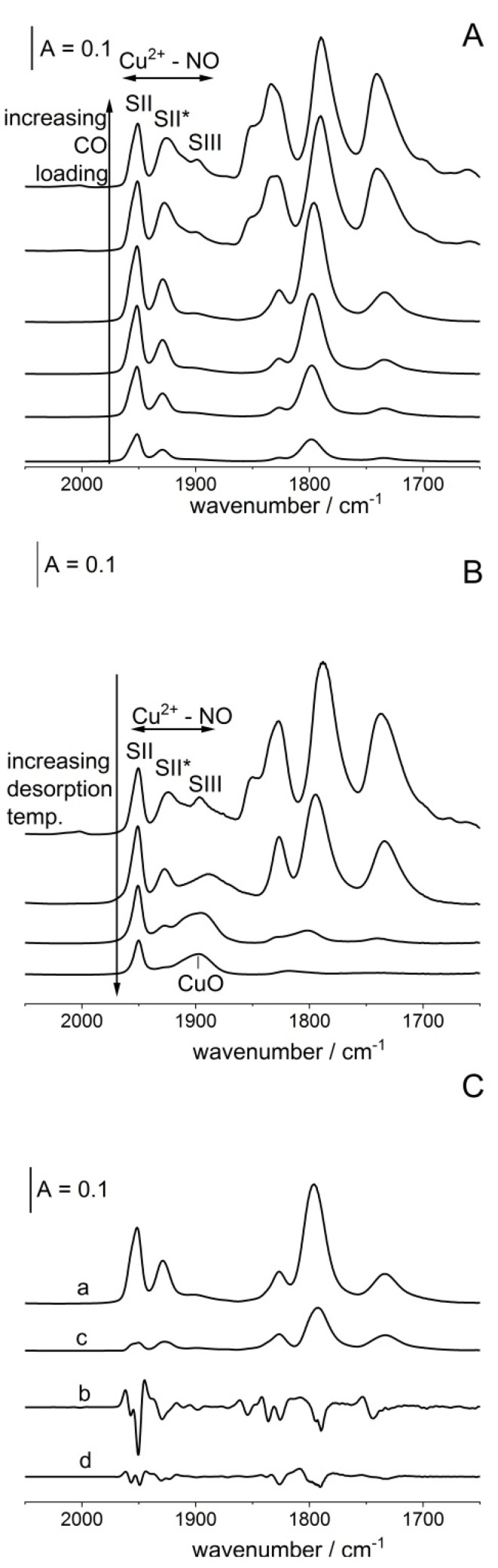
(**A**) The spectra recorded upon the sorption of increasing amounts of NO at ca. 170 K in zeolite CuY. (**B**) The spectra recorded upon the desorption of NO from CuY at vacuum in the temperature range 170–240 K. (**C**) a—the spectrum recorded upon the sorption of 10th dose of NO; b—the difference between the spectra recorded upon the sorption of 5th and 4th NO dose; c,d—the second derivatives of the spectra a and b, respectively.

The status and properties of Cu^2+^ ions in our zeolites CuY/100 (denoted also as CuY) were studied by adsorption of probe molecule NO at ca. 190 K. The spectra recorded upon the sorption of consecutive doses of NO are presented in Figure 1A. The spectra show the bands of Cu^2+^-NO adducts at 1900, 1930 and 1950 cm^−1^ as well as the band of Cu^+^-NO mononitrosyls (1790–1800 cm^−1^) and Cu^+^(NO)_2_ dinitrosyls (1735–1740 and 1825–1850 cm^−1^). These results agree with the earlier report of Palomino et al. [32], who also observed the NO bands 1923 and 1955 cm^−1^ and ascribed them to Cu^2+^-NO adducts formed by Cu^2+^ in S_II*_ and S_II_ positions. The band at 1900 cm^−1^ may be attributed to NO bonded to Cu^2+^ coordinated by four oxygen atoms (i.e., in S_III_ sites) [37]. The NO frequencies in Cu^2+^-NO adducts decrease in the order: S_II_ > S_II*_ > S_III_ (1950 > 1930 > 1900 cm^−1^); this may be explained by the fact that Cu^2+^ is the least neutralized by framework oxygens in S_II_ and the most neutralized in S_III_. Therefore, the extent of π back donation (i.e., the transfer of d electrons of cation to π* antibonding orbital of NO) increases in the order S_II_ < S_II*_ < S_III_. The varying degree of neutralization of Cu^2+^ may be related to the varying number of framework oxygens surrounding the cation and the distance between the ion and the oxygens. Cu^2+^ in S_III_ is surrounded by four oxygens, whereas the S_II*_ cation is in the plane of the equilateral oxygen triangle, and S_II_ forms a trigonal pyramid. 

The experiments in which NO adsorbed at 170 K was then desorbed by evacuation at increasing temperatures (in the range 170–240 K) were carried out. The results are presented in Figure 1B. The evacuation decreases the Cu^2+^S_III_-NO band at 1900 cm^−1^ in the first order, Cu^2+^S_II*_-NO band at 1930 cm^−1^ in the second order, and Cu^2+^S_II_ -NO band at 1950 cm^−1^ in the last order. These results suggest that the less positive Cu^2+^ (in S_III_) bonds NO molecules the most weakly but activates them the most strongly. On the other hand, the most positive Cu^2+^ (in S_II_) bonds NO strongly but activates molecules to the smallest extent. The spectra recorded upon the evacuation at 220 and 240 K show also a broad maximum at 1895–1900 cm^−1^. This maximum can be ascribed to NO interacting with CuO [38]. It suggests that some Cu^+^ ions are oxidized by NO forming CuO. A similar situation was observed in our earlier study of oxidation of Cu^+^ in CuFAU of Si/Al = 31. 

Fine analysis of Cu^2+^-NO bands suggests that these bands are complex. It is clearly seen for the Cu^2+^S_II_-NO band at 1950 cm^−1^, for which the second derivative diagram shows two minima at 1949 and 1957 cm^−1^ (Figure 1C). For the Cu^2+^S_II*_-NO band at 1930 cm^−1^ the situation is less clear. Even though this band at high NO loading does not show splitting (the second derivative diagram shows only one minimum—spectrum b in Figure 1C), the 1930 cm^−1^ band is relatively broad (the half width is comparable to a complex band at 1950 cm^−1^). The hypothesis that the 1930 cm^−1^ band is also complex is supported by the fact that the second diagram of the difference spectrum (difference between spectra recorded upon the sorption of the fifth and the fourth NO dose—spectrum c in Figure 1C) shows two minima at 1923 and 1930 cm^−1^. To sum up, it can be said that most probably both Cu^2+^-NO bands are complex and comprise two submaxima. It is clearly evident for Cu^2+^S_II_-NO and only probable for Cu^2+^S_II*_-NO. The presence of two kinds of Cu^2+^ of somewhat different positive charges and, therefore, different extents of π back donation to NO may be related to the presence of cations of various numbers of AlO_4_^−^ in close proximity. This problem will be discussed in detail later when considering the properties of Cu^+^ ions and their interaction with CO molecules. It should be mentioned, however, that the presence of two kinds of Cu^2+^ both in S_II_ and in S_II*_ positions was not reported before.

### 2.2. The Properties of Cu^+^

The properties of Cu^+^ in zeolite CuY(100) were studied with CO as probe molecules. While NO can be successively used for the studies of Cu^2+^ this molecule is less useful for the studies of Cu^+^, because Cu^+^(NO)_2_ dinitrosyls are formed together with mononitrosyls even at low NO loadings. 

**Figure 2:**
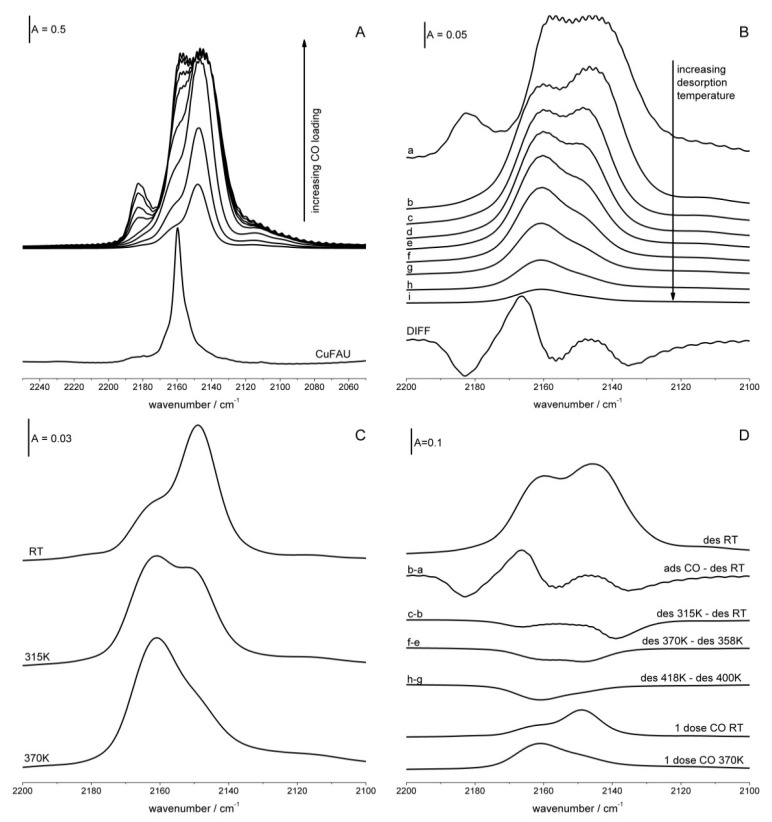
(**A**) The spectra recorded upon the sorption of increasing amounts of CO at room temperature in zeolite CuY. Bottom spectrum is the spectrum of CO sorbed in zeolite CuFAU (Si/Al = 31). (**B**) The spectrum recorded upon the sorption of CO at room temperature (top spectrum), spectra recorded upon the desorption of CO at vacuum in the temperature range 300–520 K, as well as difference between the spectra recorded upon desorption at 300 K and adsorption of CO. (**C**) The spectrum recorded upon the sorption of a small dose of CO at room temperature and heating to 315 and 370 K. (**D**) The spectrum of CO sorbed in zeolite CuY at room temperature, difference spectra recorded upon the desorption at various temperatures, and the spectrum of a small dose of CO sorbed at room temperature and upon heating to 315 and 370 K.

The spectra recorded upon the sorption of successive doses of CO in zeolite Cu(100) are presented in Figure 2A. The spectra show two distinct bands at 2147 and 2160 cm^−1^ at low CO loadings, but at higher loadings a third band at 2182 cm^−1^ appears. This last band is attributed to symmetric stretching of Cu^+^(CO)_2_ dicarbonyls. This assignment is supported by the fact that the 2182 cm^−1^ band disappears together with another dicarbonyl band at 2155 cm^−1^ upon evacuation at room temperature (Figure 2B), and a new band of Cu^+^-CO monocarbonyls at 2165 cm^−1^ grows (this is the best seen in the difference spectrum—bottom spectrum in Figure 2B). The frequencies of dicarbonyl bands (2182 and 2155 cm^−1^, respectively) are practically the same as for zeolite CuZSM-5 (e.g., [39]). According to the data presented in Figure 2B only CuS_II_ can form dicarbonyls—Cu^+^ in S_II*_ sites is unable to bond two CO molecules. It may be caused by the fact that Cu^+^ ions in S_II*_ are tightly surrounded by framework oxygens and there is not enough space to locate two CO molecules. Such location of two CO molecules is easier for S_II_ sites in which Cu is above the plane of framework oxygens on the top of the pyramid. 

The same bands of Cu^+^-CO adducts were reported by Palomino et al. [32] and were ascribed to CO interacting with Cu^+^ in S_II*_ (2147 cm^−1^) and S_II_ (2160 cm^−1^) sites. Very interesting results concerning the migration of Cu^+^ in the presence of CO were also reported by the same authors [32], who observed that Cu^+^ moved from the S_II*_ site to more exposed S_II_, reacting with three CO molecules at liquid nitrogen temperature.

The stability of Cu^+^-CO adducts was studied in desorption experiments (Figure 2B). As mentioned above, the evacuation at room temperature caused the transformation of dicarbonyls into monocarbonyls. Comparing the spectra shows that the Cu^+^S_II*_-CO adducts are less stable than Cu^+^S_II_-CO—the band at 2147 cm^−1^ decreases in the first order before the 2160 cm^−1^ one, evidencing that CO is more weakly bonded to Cu^+^S_II*_ than to Cu^+^S_II_. This conclusion agrees with that drawn from the experiments of NO desorption from Cu^2+^ sites. Cu sites of lower positive charge activate CO or NO molecules more strongly but bind these molecules more weakly. The same conclusion comes also from the experiment in which the redistribution of CO between various Cu sites was studied (Figure 2C). The small dose of CO gives the maximum at 2149 cm^−1^, and this maximum shifts to 2162 cm^−1^ upon the heating to 370 K, proving again that Cu^+^_II_ bonds CO stronger than Cu^+^_II*_. 

The bands of Cu^+^-CO are relatively large, suggesting that they are complex and composed of several submaxima, even though the fine structure of these bands is not seen. The nature of Cu^+^ sites was studied by the analysis of the difference spectra recorded upon the desorption of CO and the spectra recorded upon the sorption of a small dose of CO at room temperature followed by the heating to 370 K. These spectra are presented in Figure 2D, and the analysis of the spectra suggests that both Cu^+^_II_-CO and Cu^+^C_II*_-CO bands compose of two submaxima. The evacuation at room temperature and 315 K causes the desorption of CO from Cu^+^ in S_II*_ sites for which a CO frequency of 2137–2139 cm^−1^ is characteristic, and the evacuation at 370 K causes the desorption of CO from Cu^+^C_II*_ sites for which a CO frequency of 2149 cm^−1^ is characteristic. The adsorption of a small dose of CO at room temperature caused the rise of the 2149 cm^−1^ band. It may be concluded that two kinds of Cu^+^ ions are present in S_II*_ positions. The frequencies of CO bands for these Cu^+^ are 2137–2139 and 2149 cm^−1^. These two kinds of cations differ in the extent of activation of CO molecule, they also bond CO with different strengths. 

A similar situation is observed for Cu in S_II_ sites. The transformation of dicarbonyls to monocarbonyls causes the rise of the 2167 cm^−1^ Cu^+^S_II_ band. The adsorption of a small dose of CO followed by heating to 370 K produced a band at 2161 cm^−1^. This Cu^+^-CO species is rather stable. Only desorption at the relatively high temperature of 415 K removes CO from the sites characterized by a CO frequency of 2161 cm^−1^. Therefore, it may be concluded that two kinds of Cu^+^ sites exist in S_II_ positions. They are characterized by CO frequencies 2161 and 2167 cm^−1^. 

To sum up, it can be said that according to CO adsorption experiments in both S_II_ and in S_II*_ two kinds of Cu^+^ exist, the positive charges of which are neutralized to varying extents. It should be noted that NO sorption experiments suggest also that two kinds of Cu^2+^ in S_II_ and S_II*_ were present. As mentioned, we suppose that this may be related to the varying numbers of AlO_4_^−^ in close proximity to the Cu cation. According to ^29^Si MAS NMR results the signals of Si(0Al), Si(1Al), Si(2Al), and Si(3Al) are present in the NMR spectra of zeolites Y; the most intensive are Si(1Al) and Si(2Al) [40,41,42,43,44]. Therefore, it may be supposed that the surrounding of Cu sites in zeolites CuY may be represented as follows: (SiO)_3_Si-OCu-Al(OSi)_3_ and (SiO)_2_(AlO)Si-OCu-Al(OSi)_3_. In the second ones, Cu is more neutralized by negatively charged AlO_4_^−^. This may explain the presence of two kinds of Cu^+^ and Cu^2+^ of varying positive charges. It should be noted that the earlier IR study evidences that an analogous situation was observed for Si-OH-Al groups in zeolites Y. Several kinds of such hydroxyls of various acidity, i.e., of various positive charge on hydrogen, were found and it was explained by the presence of a varying number of AlO_4_^−^ in close proximity [44]. The dependence of CO stretching frequency in Cu^+^-CO adducts was a subject of quantumchemical calculations of Rejmak et al. [45]. It is important to mention that the heterogeneity of Cu^+^ sites in both S_II_ and S_II*_ positions was not reported before. 

The properties of Cu^+^ ions in zeolite CuY (Si/Al = 2.5) and in CuFAU (Si/Al = 31) can be compared. According to the data presented in Figure 2A the band of Cu^+^-CO in zeolite CuFAU is narrower than that in CuY, evidencing homogeneity of Cu^+^ sites in CuFAU. Similarly, Si-OH-Al groups are also homogeneous (all express the same acid strength) in HFAU [46,47]. In this highly siliceous zeolite, all the Cu^+^ ions and all the hydroxyls have the same number of AlO_4_^−^, i.e., only one AlO_4_^−^, in close proximity. The frequency of Cu^+^-CO adducts in CuY and CuFAU can be compared in Figure 2A. The Cu^+^-CO frequency for CuFAU is 2158 cm^−1^. The same frequency was reported for another highly siliceous CuZSM-5 [39]. For zeolite CuY Cu^+^ in S_II*_ sites the Cu^+^-CO frequency is 2137–2139 and 2149 cm^−1^, i.e., are lower than for CuFAU (2158 cm^−1^). This may be explained by a higher number of AlO_4_^−^ in CuY than in CuFAU. The question may be asked, why does Cu in S_II_ sites in CuY show a higher frequency of Cu^+^-CO (2161, and 2167 cm^−1^) than for CuFAU (2158 cm^−1^), even though they have more AlO_4_^−^ in close proximity? It is possible that the lower neutralizing effect of framework oxygens on the cation may be due to the longer distance from the cation to framework oxygens. While Cu^+^ in S_II*_ is located in the center of an oxygen ring tightly surrounded by oxygens, the Cu^+^ in S_II_ is above the ring, on the top of the trigonal oxygen pyramid. The longer distance causes less effective neutralization of the cation and higher Cu^+^-CO frequency.

### 2.3. Reduction of Cu Ions

**Figure 3:**
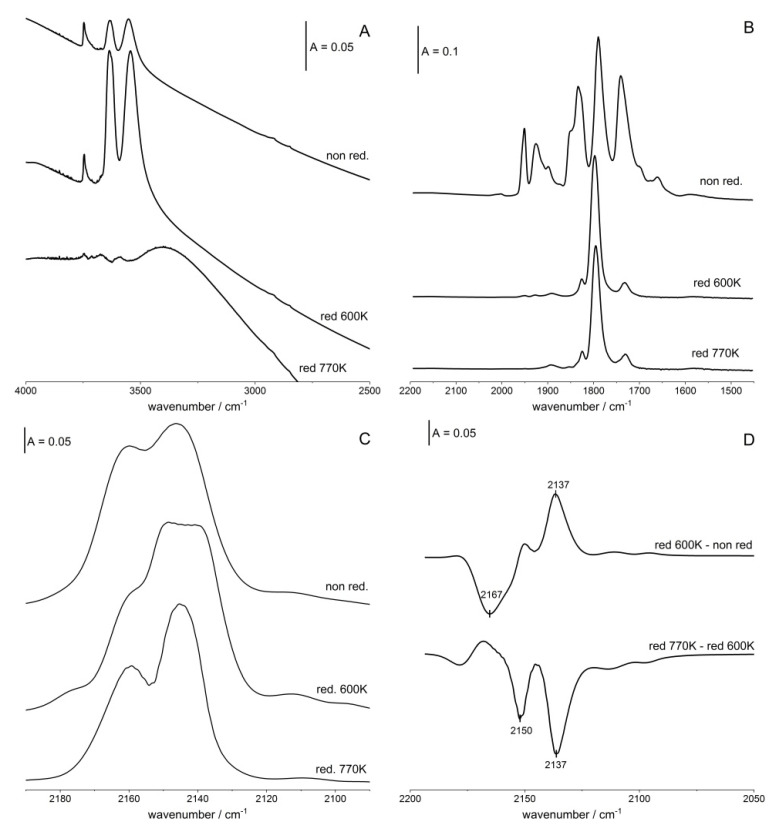
(**A**) The spectra of OH groups in zeolite CuY non-reduced and in zeolite reduced with hydrogen at 600 and 770 K. (**B**,**C**) The spectra of NO (**B**) and CO (**C**) sorbed at 170 K (NO) and room temperature (CO) in zeolite CuY non-reduced and reduced at 600 and 770 K, (**D**) Difference spectra: differences between the spectra recorded upon the reduction at various temperatures.

The reduction of Cu ions was carried out with hydrogen at 600 and 770 K followed by evacuation at 570 K. The spectra of OH groups in non-reduced and reduced zeolites are presented in Figure 3A. The spectrum of non-reduced CuY shows two distinct Si-OH-Al bands at 3550 and 3630 cm^−1^ of Si-O_3_H-Al and Si-O_1_H-Al. These hydroxyls were formed by the hydrolysis: Cu^2+^ + H_2_O = CuOH^+^ + H^+^. CuOH^+^ undergoes further reactions, finally producing Cu^+^ (evidenced by CO adsorption), and H^+^ reacts with framework oxygens, forming acidic Si-OH-Al. The reduction with hydrogen at 600 K significantly increases the intensity of Si-OH-Al bands, evidencing the formation of new acidic hydroxyls. This may be explained by considering the process in which Cu ions (Cu^2+^ and Cu^+^) decrease oxidation state and hydrogen is transformed into H^+^ (and, therefore, to Si-OH-Al). The treatment with hydrogen at 770 K causes the vanishing of Si-OH-Al bands, indicating the dehydroxylation which occurs at high temperatures. The dealumination and destruction of CuY zeolite upon reduction were observed by Petunchi et al. [48].

The spectra of Cu^2+^-NO adducts are presented in Figure 3B. The treatment with hydrogen at a relatively low temperature of 600 K reduces practically all the Cu^2+^—the bands at 1930 and 1950 cm^−1^ vanished. These results evidence that Cu^2+^ ions in zeolite CuY are more prone to reduction than Cu^+^—the Cu^+^-CO bands are still very intense (Figure 3C). Great susceptibility of Cu^2+^ in zeolites for reduction was already observed for zeolites CuFAU of Si/Al = 31 [36]. 

**Figure 4:**
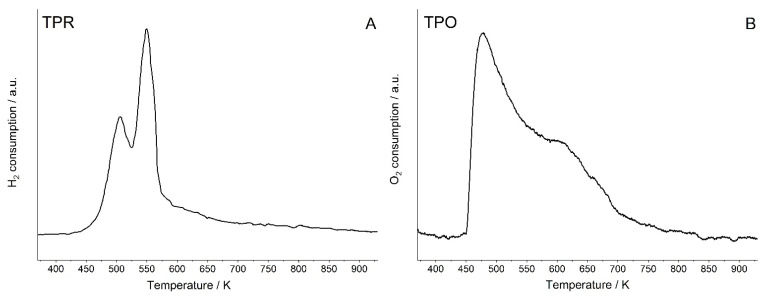
Temperature-programmed reduction (TPR) (**A**) and oxidation (TPO) (**B**) diagrams of CuY.

Further information on the reduction of both Cu^2+^ and Cu^+^ in CuY was obtained in the experiments of CO sorption (Figure 3C,D). The reduction at 600 K causes the decrease in the Cu^+^-CO maximum at 2167 cm^−1^ and an increase in the maximum at 2137 cm^−1^. As mentioned, according to the data presented in Figure 4B reduction at 600 K causes the disappearance of Cu^2+^-NO bands at 1930 and 1950 cm^−1^. It may be, therefore, supposed that the treatment with hydrogen at 600 K causes the reduction of Cu^2+^ ions, with the formation of Cu^+^ characterized by the Cu^+^-CO band at 2137 cm^−1^, i.e., Cu^+^ in S_II*_ sites which is the least positive. Simultaneously the treatment with hydrogen at 600 K causes the decrease in the Cu^+^-CO band at 2167 cm^−1^. We think that this is due to a reduction of these Cu^+^ ions to Cu^0^. To sum up, it can be said that treatment with hydrogen at 600 K reduces all Cu^2+^, producing the less positive Cu^+^ in S_II*_. Simultaneously, the most positive Cu^+^ ions in S_II_ are reduced to metallic Cu. 

The treatment with hydrogen at a higher temperature (770 K) causes the further reduction of Cu^+^—the Cu^+^-CO bands at 2161 and 2137 cm^−1^ diminish (Figure 3D). The reduction of both Cu^+^ and Cu^2+^ ions should produce Cu^0^, characterized by a Cu^0^-CO band at 2050–2090 cm^−1^. However, this band is absent in the spectra of CO adsorbed (Figure 3D). It is not excluded that the agglomerates of metallic Cu are located inside cubooctahedra, are, therefore, not accessible to CO molecules. 

Complementary to IR studies of reduction of Cu species, the TRP (temperature-programmed reduction) experiments were carried out. The results presented in Figure 4A evidence that the reduction of Cu species occurs in two steps; this agrees with our IR results, which showed that more positive Cu species (Cu^2+^ and Cu^+^ characterized by Cu^+^-CO band 2167 cm^−1^) are reduced at lower temperatures than less positive Cu^+^ ones. It is possible that low temperature TPR maximum concerns the reduction of such more positive Cu ions. 

### 2.4. Oxidation of Cu Sites

**Figure 5:**
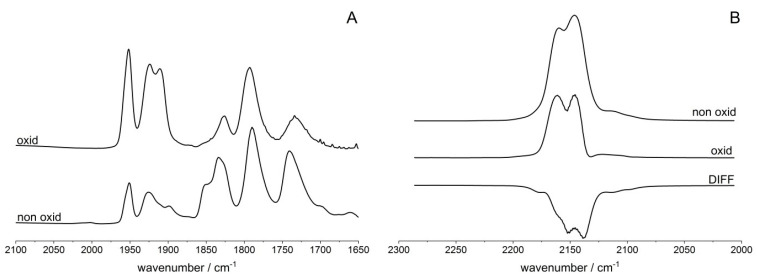
The spectra of NO sorbed at 170 K (**A**) and CO sorbed at room temperature (**B**) in zeolite CuY non-oxidized and oxidized at oxygen at 570 K.

The oxidation of Cu sites in zeolite CuY was achieved by the treatment with oxygen at 570 K followed by evacuation at the same temperature. The spectra of NO sorbed in zeolites (Figure 5A) show that oxidation causes the formation of Cu^2+^ sites in exchange positions characterized by Cu^2+^-NO bands at 1950 cm^−1^ (S_II_ sites) and 1925 cm^−1^ (S_II*_ sites). It may be supposed that the 1910 cm^−1^ band may be attributed to the NO bonded to Cu^2+^ in S_III_ sites. 

The significant increase in the amount of Cu^2+^ may be related to the loss of Cu^+^ (Figure 5B). The decrease of Cu^+^-CO bands is evident. The analysis of the difference spectrum shows that Cu^+^ in S_II*_ sites (Cu^+^-CO band 2137 cm^−1^), i.e., the cations which are the less positive are the most prone to oxidation. These cations have the greatest tendency to be electron donors to oxygen atoms, increasing their oxidation state.

Complementary to IR experiments the TPO (temperature-programmed oxidation) study was conducted. The results presented in Figure 4B evidence that oxidation occurs in two steps, which agrees with IR data. The oxidation of less positive Cu^+^ takes place at a lower temperature. 

### 2.5. Effect of Cu Content

In order to follow the effect of Cu content in zeolite CuY on the properties of Cu sites, NO and CO were sorbed in zeolites of exchange degrees 100% and 45%. 

**Figure 6:**
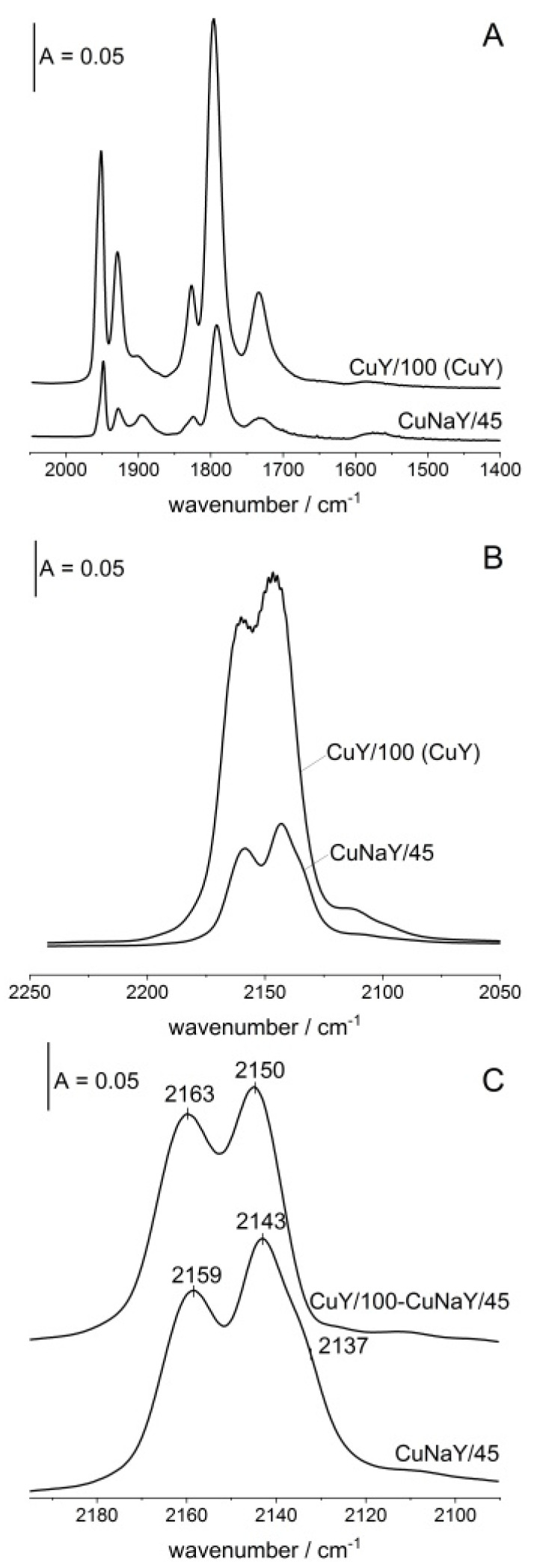
(**A**,**B**) The spectra of NO (**A**) and CO (**B**) sorbed at 170 K (NO) and at room temperature (CO) in zeolites of exchange degrees 100% and 45%. (**C**) Differences between the spectra of CO sorbed in zeolites of various Cu contents. Spectra are normalized to the same band intensity.

The spectra of NO sorbed at ca. 170 K are presented in Figure 6A. The bands of Cu^2+^-NO at 1930 and 1950 cm^−1^ increase with Cu content, evidencing the increase in Cu^2+^ amount.

More interesting results concern Cu^+^ ions. The spectra of CO sorbed at room temperature in both zeolites are presented in Figure 6B and the difference spectrum is shown in Figure 6C. The spectrum of Cu^+^-CO in zeolite of exchange degree 45% is shown too. This spectrum concerns Cu^+^ introduced in the first step. The difference spectrum 100% minus 45% concerns Cu^+^ ions introduced in the next step (at higher Cu contents). Both spectra presented in Figure 6C are normalized to the same intensity of Cu^+^-CO band. The increase in Cu contents results in a shift of both Cu^+^S_II_ and Cu^+^S_II*_ bands to higher frequencies, suggesting that the first introduced Cu creates Cu^+^, which is the most neutralized by framework oxygens, i.e., situated in sites having a higher number of AlO_4_^−^ tetrahedra. Such Cu cations are the most stabilized by framework oxygens.

## 3. Materials and Methods

### 3.1. Materials

The parent Na-Y zeolite was synthesized according to instructions in [49]. Framework Si/Al calculated from ^29^Si MAS NMR results was 2.5. Ion exchange was performed by mixing zeolite powder of Na-Y with water solution of CuNO_3_. The sample of exchange degree 100% (denoted as CuY/100 or simply CuY) was prepared using 4 g of zeolite powder and 160 mL of Cu solution (0.5 mol/dm^3^) by mixing at 80 °C for 2 h. The ion exchange was repeated 4 times with centrifugation of the sample after each exchange. The sample of the exchange 45% (denoted as CuNaY/45) was prepared by treatment of 2 g of zeolite with 175 mL of 0.01 mol/dm^3^ of CuNO_3_ for 1 h. After the ionic exchange procedure, the samples were washed with distilled water and centrifuged 4 times. Finally, the samples were dried overnight in 75 °C. The content of Cu was determined by XRF analysis.

### 3.2. XRF Studies

XRF spectroscopy was used to determine the wt.% of Cu in the prepared samples. The measurements were performed using the EDX 3600H apparatus by Skyray Instrument Inc. (Stoughton, MA, USA) equipped with a tungsten lamp of 40 kV voltage. The copper content was calculated based on the calibration curve prepared for the mixture of CuO and Na-Y. It was 10.56 and 4.88 wt.% of Cu, which corresponded to 100% and 45% of exchange (calculated as Cu/2Al). As mentioned above, these samples were denoted as CuY/100 and CuNaY/45, respectively. 

### 3.3. IR Studies

Prior to IR experiments, zeolites were evacuated in situ in an IR cell at 720 K for 1 h. The spectra were recorded with a NICOLET 6700 spectrometer (Thermo Scientific, Cambridge, MA, USA) with the spectral resolution of 1 cm^−1^. CO and NO (Air Products, Allentown, PA, USA) were used as probe molecules. The adsorption of CO was performed at room temperature. Adsorption of NO was performed at ca. 190 K. The doses of CO and NO, each corresponding to ca. 5–10% of Cu content, were adsorbed. 

### 3.4. Temperature-Programmed Reduction 

Temperature-programmed reduction with hydrogen (H_2_-TPR) was carried out on a Chembet-3000 (Quantochrome, Boynton Beach, FL, USA). The hydrogen consumption was monitored with a TCD detector. For the typical H_2_-TPR experiment, sample (25 mg) was placed in a quartz U-shape tube reactor and activated at 370 K in He flow (30 mL/min) for 1.5 h. Next, the sample was cooled down to RT in He flow and the H_2_-TPR experiment was performed in 5% H_2_/Ar (30 mL/min) in the temperature range RT—920 K (∆T = 10 K/min).

### 3.5. Temperature-Programmed Oxidation

Temperature-programmed oxidation measurement (TPO) was performed in the quartz fixed-bed flow reactor connected online to a mass spectrometer (QMG 220 PRISMA PLUS). Prior to TPO run, sample (50 mg) was activated in the stream of He (30 mL/min) at 720 K for 1 h. Next, the reactor was cooled down to room temperature (RT) and TPO was carried out in the stream of 5% O_2_/He (30 mL/min) from RT to 920 K with ∆T = 10 K/min. During TPO, signal *m*/*z* = 32 (O_2_) was monitored.

## 4. Conclusions

The properties of both Cu^2+^ and Cu^+^ in zeolites CuY were studied with NO and CO as probe molecules. It was found that Cu^2+^ ions were located in S_II_, S_II*_, and S_III_ sites. The less positive Cu^2+^ (in S_III_) binds NO molecules the most weakly but activates them the most strongly. On the other hand, the most positive Cu^2+^ (in S_II_) binds NO strongly but activates this molecule to the smallest extent. Cu^+^ is located in S_II_, S_II*_. Fine analysis of the spectra of the adsorbed NO and CO suggested that both in S_II_ and S_II*_ of Cu two kinds of Cu ions of different positive charges are present. It may be supposed that these Cu ions have various numbers of AlO_4_^−^ in close proximity, and that their positive charge is neutralized by the framework oxygens to varying extents. The experiments of reduction with hydrogen evidenced that the susceptibility of Cu sites to reduction increases as the positive charge on the cation increases. Therefore, the most prone are Cu^2+^ and also Cu^+^, which are less neutralized by oxygens, and the least prone to reduction are the less positive Cu^+^ ions (i.e., the most neutralized by framework oxygens). It may be supposed that more positive Cu ions have a stronger tendency to attract electrons, which causes the lowering of the oxidation state. The reduction of CuY with hydrogen produces acidic Si-OH-Al groups. An opposite tendency was observed in oxidation experiments. The susceptibility of Cu sites to oxidation decreases with a positive charge on Cu^+^. The less positive Cu^+^ ions are the most active in a donation of electrons to oxygen atoms, which increases the oxidation state of Cu. The experiments with CuNaY zeolites of various Cu contents suggest that the first introduced Cu (at low Cu contents) created Cu^+^, which was the most neutralized by framework oxygens. Such Cu cations are the most stabilized by framework oxygens. 

## Data Availability

Not applicable.

## Sample Availability

Samples of the compounds of CuY zeolites are available from the authors.
